# Comparison between next-generation and Sanger-based sequencing for the detection of transmitted drug-resistance mutations among recently infected HIV-1 patients in Israel, 2000–2014

**DOI:** 10.7448/IAS.20.1.21846

**Published:** 2017-08-10

**Authors:** Roy Moscona, Daniela Ram, Marina Wax, Efrat Bucris, Itzchak Levy, Ella Mendelson, Orna Mor

**Affiliations:** ^a^ Central Virology Laboratory, Ministry of Health, Sheba Medical Center, Ramat-Gan, Israel; ^b^ Infectious Disease Unit, Sheba Medical Center, Ramat-Gan, Israel; ^c^ School of Public Health, Tel Aviv University, Ramat-Aviv, Israel

**Keywords:** HIV-1, recently infected, transmitted drug-resistance mutations, next-generation sequencing, Sanger-based sequencing, DeepChek-HIV.

## Abstract

**Introduction**: Transmitted drug-resistance mutations (TDRM) may hamper successful anti-HIV-1 therapy and impact future control of the HIV-1 epidemic. Recently infected, therapy-naïve individuals are best suited for surveillance of such TDRM. In this study, TDRM, detected by next-generation sequencing (NGS) were compared to those identified by Sanger-based population sequencing (SBS) in recently infected HIV-1 patients.

**Methods**: Historical samples from 80 recently infected HIV-1 patients, diagnosed between 2000 and 2014, were analysed by MiSeq (NGS) and ABI (SBS). DeepChek-HIV (ABL) was used for interpretation of the results.

**Results**: Most patients were males (80%); Men who have sex with men (MSM) was the major transmission group (58.8%). Overall, TDRM were detected in 31.3% of patients by NGS and 8.8% by SBS, with SBS TDRM restricted to persons infected with subtype B. All SBS-detected TDRM were identified by NGS. The prevalence of TDRM impacting protease inhibitors (PI), nucleoside reverse transcriptase inhibitors (NRTI) and non-nucleoside reverse transcriptase inhibitors (NNRTI) was 11.3, 26.2 7.5%, respectively, in NGS analyses and 0, 3.8 and 5%, respectively, in SBS analyses. More patients with NGS and SBS TDRM were identified in 2008–2014 (37.2% or 13.9%, respectively) compared to 2000–2007 (24.3% or 2.7%, respectively), and a significantly greater number of these patients had multiple NGS TDRM. The most abundant, albeit, minor-frequency RT TDRM, were the K65R and D67N, while K103N, M184V and T215S were high-frequency mutations. Minor TDRM did not become a major variant in later samples and did not hinder successful treatment.

**Conclusions**: NGS can replace SBS for mutation detection and allows for the detection of low-frequency TDRM not identified by SBS. Although rates of TDRM in Israel continued to increase from 2000 to 2014, minor TDRM did not become major species. The need for ongoing surveillance of low-frequency TDRM should be revisited in a larger study.

## Introduction

Transmitted drug-resistance mutations (TDRM) challenge HIV-1 treatment of therapy-naïve individuals. They reduce therapeutic options, increase the risk for treatment failure and maintain onward transmission of drug resistance [1]. TDRM are used in HIV-1 surveillance programmes as a tool for comparison and global monitoring of transmitted protease (PR) and reverse transcriptase (RT) HIV-1 resistance [2].

Different world regions are classified as low (<%5), medium (5–15%) or high (>15%) TDRM prevalence zones [3]. The European SPREAD programme that monitors PR and RT TDRM prevalence in Western and Eastern Europe and in Israel, has reported an overall medium prevalence of 8–10% between the years 2002 and 2010 [1]. Monitoring TDRM is best pursued in samples taken soon after HIV-1 infection. Indeed, a slightly higher TDRM prevalence was reported by SPREAD for recently infected patients (10.1%) compared to patients with an unknown duration of infection (8.2%). Prevalence of nucleoside RT inhibitors (NRTI), non-nucleoside RT inhibitors (NNRTI) and protease inhibitors (PI) TDRM in 2008–2010 in this subgroup of patients was 4.7, 3.8 and 2.4%, respectively, compared to 4.4, 2.9 and 2%, respectively, in the group of patients with an unknown duration of infection [1].

TDRM frequency may vary in the viral pool, with those that are less fit identified only at a low frequency in the viral quasispecies [4]. Sanger-based sequencing (SBS), the most common method used to detect all HIV-1 resistance mutations including TDRM [5], is limited in its sensitivity, enabling the identification of mutations only if present in 15–20% of the viral population [6]. Thus, variants that may be present in a minor proportion of the viral quasispecies, are missed. Next-generation sequencing (NGS), developed in recent years, enables the detection of low- as well as high-frequency variants and was shown to be suitable for the identification of HIV-1 drug-resistance mutations (DRMs) [7]. NGS analysis of blood samples taken from treatment-naïve patients soon after HIV-1 infection could be the most appropriate means of assessing the incidence and prevalence of TDRM. Indeed, TDRM prevalence was high in acutely infected treatment-naïve HIV-1-infected persons and reduced in chronically infected individuals. In addition, some of the TDRM were identified by NGS only [8].

By 2015, 9,720 individuals were estimated to be HIV-1 positive in Israel [9]. Confirmation of HIV-1 infection is centralized in the National HIV Reference Laboratory (NHRL), that performs the confirmatory assays [10,11] and holds the national registry of infected patients. Changes in HIV-1 incidence have occurred in Israel over the years. Immigration waves from Ethiopia (mainly at 1991 and between 1998 and 2007) and the former Union of Soviet Socialist Republics (USSR, starting in 1990), raised HIV-1 incidence [12]. Refugees from Africa have also contributed to the increased number of HIV-1-infected persons in Israel. Most of these individuals were infected in the country of origin and entered Israel with established, untreated HIV-1 infection. One-third of all new infections in 2014 and 2015 were men having sex with men (MSM), characterized by infection with subtype B viruses [9]. Subtype C was predominant (89%) in people emigrating from Africa, considered as those originating from counties with generalized HIV epidemics (OGE). Subtype A was prevalent (71%) among HIV-1-infected persons coming from the former USSR [13].

In this study, we screened the NHRL database for recently infected therapy-naïve patients identified during seroconversion. Recently infected was defined as individuals presenting with a change in the results of the laboratory confirmation assay from negative to positive, within a period of less than 6 months. The prevalence of HIV-1 TDRM and other RT and PR amino acid substitutions (including DRM defined by Stanford HIVDR) was assessed by NGS and SBS, in samples from 80 such patients. In addition, when possible, the clinical characteristics and treatment outcome of patients with and without TDRM were compared.

## Methods

### Study participants

Since 2000, the algorithm for diagnosis of HIV-1 infection has included initial screening, using two different third- or fourth-generation enzyme immunoassays and confirmation of reactive samples, with the Line-Immuno-Assay (LIA, InnoLia HIV I/II, Fujirebio, Ghent, Belgium). In cases with negative or indeterminate LIA results, a new sample taken at least 2 weeks after the first sample, is retested with LIA. Only LIA-positive samples can confirm HIV-1 infection. The NHRL database was screened for recently infected, therapy-naïve patients diagnosed after seroconversion, defined as a negative or indeterminate LIA test less than 6 months before a positive LIA. Patients with LIA-defined seroconversion, from whom sufficient material (or serum stored in −80 °C) was still available, were included in this study (*n* = 80). The study was approved by the ethical committee of the Sheba Medical Center (1765–14-SMC). Being a retrospective study, which is based on anonymous archived data, informed consent was not requested.

### Extraction of HIV-1 RNA and amplification of target regions

Ribonucleic acids (RNA) were extracted from 0.5 mL plasma (or serum) samples by NucliSENS Easy MAG (Biomerieux, Marcy l’Etoile, France), according to the manufacturer’s protocol, and eluted in a final volume of 55 µl. A 1.8-kb fragment containing HIV-1 PR and RT, was generated by RT-polymerase chain reaction (RT-PCR), followed by nested PCR, according to published protocols [14,15]. A construct containing wild-type HIV-1 (HIV-1 pLAI) was also amplified and used as control for the amplification and NGS analysis [16].

### SBS and NGS sequencing and statistical analysis

Nested PCR fragments corresponding to the PR and RT of HIV-1, were subjected to SBS, performed using the ABI Analyzer 3100 (Thermo Fisher, Waltham, MA). The same fragments were used to prepare paired end indexed libraries with Nextera DNA sample preparation kit (Illumina, San Diego, CA). These were subsequently run on the MiSeq instrument (Illumina, San Diego, CA). DeepChek-HIV TherapyEdge17 version 1.1 (ABL, Luxemburg) was used for automated sequencing analysis and resistance interpretation of both SBS and NGS results. HXB2 was used as a reference strain. Amino acid positions 4–99 in the PR and positions 40–247 in the RT (which cover all resistance mutations) in all the tested samples were selected for further analysis. TDRM were defined according to the WHO list of surveillance DRMs, and RT and PR DRM were according to the Stanford HIVDR Database [2,17]. A threshold of 1.5% prevalence for any mutation was set based on the analysis of pLAI control, in which all identified mutations were considered artifactual [supplement 1] and were all observed at a frequency below 1.5%. The average number of reads covering the PR and RT was 4,234 (IQR 2989–5074) and 4,249 (IQR 2817–5246), respectively. In all Miseq runs, 8 × 10^5^ to 1 × 10^6^ reads passed quality assurance, with Q-scores ≥Q30. Statistical analyses were performed with SPSS version 21 (IBM, North Castle, NY, USA). For continuous variables, a T-test was performed, and for categorical variables, a Fisher’s exact or Mcnemar’s test (for paired data analysis) were used. In all tests, two-sided *p*-values *p* < 0.05 were considered significant. All sequences were submitted to the NCBI GeneBank (study SRP105689).

## Results

### Characterization of study population

Between January 2000 and December 2014, 5830 HIV-1-infected persons were identified by the NHRL in Israel, 4.1% (236/5830) of whom were diagnosed during seroconversion. Among this subpopulation, 84% (199/236) were men, of a mean age of 33 (IQR 27–43); 60% (141/236) were MSM. A significantly greater number of these recently infected individuals, were identified between 2008 and 2014 (48.0%, 151/3144) as compared to 2000–2007 (31.6%, 85/2686, *p* < 0.05). Sample material from 80 of these 236 seroconverters, which represent 33.9% of all identified recently infected therapy-naïve individuals, was available for this study. 80% (64/80) of these recently infected patients were men, 58.8% (47/80) were MSM. The patients were characterized by a distinct LIA pattern of anti-HIV-1 IgG antibodies. Only 22.5% (18/80), 61.5% (50/80) and 53.8% (43/80) had acquired antibodies against the p31 (HIV-1 integrase protein), p17 matrix protein and 120 surface glycoprotein, respectively, corroborating the recent seroconversion of these individuals. NGS and SBS analyses were conducted on samples taken 19 days (median, IQR 0–27) after the LIA-positive sample. The median HIV-1 viral load and CD4 counts were 5.5 log copies/mL (IQR 4.6–6.2) and 405 cells/mm3 (*n* = 50, IQR 310–579), respectively. Most patients (60%, 48/80) carried HIV-1 subtype B, 21.2% carried subtype C and 13.7% were infected with subtype A viruses (Table 1).

**Table 1.** Characteristics of the study population (*n* = 80)

Characteristic	
Male, *n* (%)	64 (80.0)
Age, years, median (IQR)	30.5 (18–45)
Viral load (log10 copies/ml), median (IQR)	5.5 (4.6–6.2)
CD4 (cells/mm3), median (IQR) (*n* = 50)	405 (310–579)
Risk group, *n* (%)	
MSM	47 (58.8)
OGE	16 (20.0)
Other*	17 (21.2)
WB reactive bands, *n* (%)	
p17	50 (61.5)
p24	76 (95.0)
p31	18 (22.5)
gp41	97.5 (78.0)
sgp120	45 (56.3)
Time between diagnosis and resistance analysis, days (IQR)	19 (0–19)
HIV subtype, *n* (%)	
B	48 (60.0)
C	17 (21.2)
A	11 (13.7)
Other	4 (5.1)

MSM – men who have sex with men; OGE – originating in countries with generalized epidemics.

*Other subtypes- AG/G (3) and F (1).

### Protease and RT mutation prevalence, as determined by NGS and SBS

A significantly higher number of non-synonymous amino-acid changes in the RT and PR were identified in the 80 samples by NGS (PR = 719, RT = 974, with prevalence ≥1.5%) as compared to SBS (PR = 600, RT = 667, *p* < 0.05). TDRM were identified in 31.3% (25/80) and 8.8% (7/80) of patients by NGS and SBS, respectively (*p* < 0.05). The prevalence of patients with NGS-identified TDRM was 11.3% for PI, 26.3% for NRTI and 7.5% for NNRTI. Compared to NGS, a significantly lower percentage of patients harbouring TDRM was found by SBS for PI and NRTI TDRM (0% and 3.8%, respectively, *p* < 0.05) and slightly lower for NNRTI TDRM (3.8%, Table 2).

**Table 2.** Number of patients with TDRM identified by SBS and NGS

	NGS	SBS	*P*-value
Any TDRM, *n* (%)	25 (31.3)	7 (8.8)	<0.05
PI TDRM, *n* (%)	9 (11.3)	0 (0)	<0.05
NRTI TDRM, *n* (%)	21 (26.3)	4 (5.0)	<0.05
NNRTI TDRM, *n* (%)	6 (7.5)	3 (3.8)	0.53

TDRM – transmitted drug resistance mutations; PI – protease inhibitors; NRTI – nucleoside reverse transcriptase inhibitors; NNRTI – non-nucleoside reverse transcriptase inhibitors; SBS – Sanger-based sequencing; NGS – next-generation sequencing.

**Figure 1.**
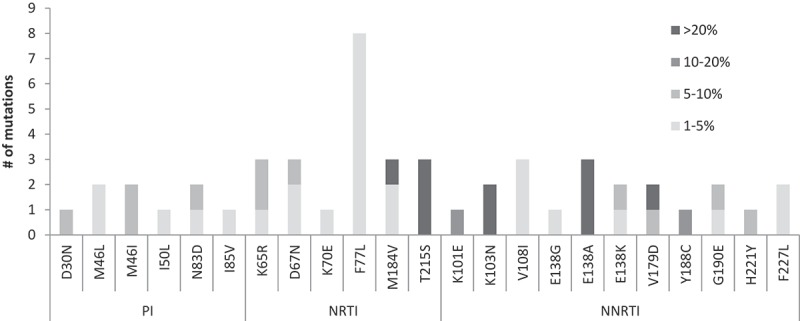
**Mutations (TDRM and DRM) according to threshold of NGS frequency (<5%, 5**–**10%, 10.1–20%, >20%) and drug class.** PI – protease inhibitors; NRTI – nucleoside reverse transcriptase inhibitors; NNRTI – non-nucleoside reverse transcriptase inhibitors; NGS – next-generation sequencing; TDRM – transmitted drug resistance mutations; DRM – drug resistance mutations.

Table 3 outlines the characteristics of all patients with TDRM and the frequency and type of TDRM identified. All SBS-identified TDRM were identified as major variants (>20% frequency) by NGS. When considering all TDRM identified by NGS, the PI mutations which were identified by NGS only, were all low-frequency variants (<10% of the quasispecies), identified in one or two patients only. M46I/L, potentially conferring low-level resistance to any PI, was the only mutation found in 5% (4/80) of the patients. The NRTI TDRM identified at low frequency by NGS (<10% of the viral population), were D67N, K65R, and K70E, as well as F77L, which was the most frequent (8 patient samples) low-abundance (1.5–5%) NRTI TDRM identified. However, since F77L is a transition mutation located within a homopolymeric region, a mutation that was also observed at a frequency below the quality assurance threshold (<1%) in 25% (18/72) of all other samples, we considered it a technical artefact of the PCR and sequencing procedures [18]. M184V (identified as minor variant in two patients) and T215S were the only highly prevalent NGS and SBS NRTI TDRM identified. While G190E and Y188C were minor NNRTI TDRM identified by NGS, K101E and K103N were identified by both NGS and SBS. Few polymorphic resistance mutations were identified by both NGS and SBS, e.g., V179D and different substitutions of the NNRTI DRM E138 amino acid. Mainly, E138A predicted to mostly affect rilpivirine resistance, was identified in three patients (infected with subtype A, AG and C) by both sequencing platforms (Figure 1).

**Table 3.** Patients with TDRM identified by NGS and SBS

Patient number	Year of diagnosis	Risk group	VL log (c/ml)	CD4 (cells/mm3)	Virus subtype	NGS TDRM (prevalence, %)			SBS TDRM		
						PI	NRTI	NNRTI	PI	NRTI	NNRTI
24999	2002	OGE-IL	6.3	UKN	C	_	M184V(1.6)	_	_	_	_
26203	2002	OGE-IL	4.9	220	C	_	K65R(1.5)	_			
27054	2002	OGE-IL	5.5	230	C	N83D(5.5)	F77L(1.6)	_	_	_	_
28751	2003	MSM	4.3	355	B	I85V(1.9)	_	_	_	_	_
29689	2003	MSM	4.9	356	B	_	F77L(1.7)	_	_	_	_
29704	2003	OGE-IL	5.9	UKN	C	_	D67N(4.4)	_	_	_	_
31113	2003	MSM	5.6	UKN	B	_	T215S(99.7)	_	_	T215S	_
33020	2004	MSM	2.3	616	B	_	D67N(2.9)	_	_	_	_
47845	2007	MSM	3.6	519	B	_	_	G190E(8.4)	_	_	_
53814	2009	MSM	3.9	UKN	B	_	K65R(12.4)	_	_	_	_
54236	2009	MSM	3.5	517	B	_	F77L(2.5)	_	_	_	_
56365	2010	MSM	5.6	998	B	M46L(2.3)	T215S(99.1)	_	_	T215S	_
63124	2011	MSM	4.3	UKN	A	I50L(3.2)	_	_	_	_	_
63729	2011	OGE-IL	3.5	811	C	_	K65R(2.5)	_			
63942	2011	MSM	4.6	665	B	_	M184V(2.2)	_	_	_	_
64821	2011	MSM	4.9	UKN	B	_	K70E(2.2)	_	_	_	_
66012	2012	MSM	5.7	341	B	_	F77L(1.9)	K103N(98.9)	_	_	K103N
66122	2012	MSM	5.2	427	A	_	F77L(1.9)	_	_	_	_
66655	2012	IDU	6.7	290	A	N83D(3.9)	F77L(1.7)	_	_	_	_
66844	2012	MSM	6.8	431	B	M46I(6.6)	T215S(96.7)	G190E(1.5)	_	T215S	_
67245	2012	Other	5.9	UKN	A	D30N(6.6)	D67N (6.9)		_	_	_
68428	2012	MSM	4.7	400	B	_	F77L (2.1)	Y188C(10.7)	_	_	_
72140	2014	Hetero	4.6	UKN	B	M46L(1.6)	M184V(96.6), F77L (1.5)	_	_	M184V	_
72156	2014	Other	5.9	UKN	B	_	_	K103N(97.2)	_	_	K103N
72675	2014	Other	5.93	27	B	M46I(5.6)	_	K101E (23.3)	_	_	K101E

Some of the low-frequency TDRMs identified are consistent with APOBEC-mediated G-to-A editing: the PI M46L/I and D30N, the NRTI D67N and the NNRTI G190E TDRM, as well as the E138K amino substitution, were all related to APOBEC [19]. It was suggested that NGS may artificially present such mutations and that in treatment-naïve patients, sequences that display several such TDRM, should be carefully examined [20]. While in almost all patients, these APOBEC-related mutations were sporadic, in two of the patients (66,844 and 67,245), several such mutations were observed together (Table 3), questioning their clinical significance.

### Characterization of recently infected patients with and without TDRM

Table 4 compares the characteristics of patients with and without TDRM, as identified by NGS and SBS. Recently infected individuals with NGS-identified TDRM had significantly lower viral load (median of 4.9 versus 5.8 log copies/ml, *p* < 0.05) and a higher number of RT (but not of PR) non-synonymous amino acid substitutions (mean of 12.6 versus 9.7 amino acid changes, *p* < 0.05), as compared to those without NGS-identified TDRM. Although their number was higher, these NGS-identified substitutions were, on average, of lower frequency in the viral quasispecies compared to the average frequency of all amino acid substitutions identified in patients without TDRM (average frequency of 53% versus 77%, *p* < 0.05) and most were below 5% frequency. SBS-identified (but not NGS-identified) TDRM were restricted to subtype B-infected persons (*p* < 0.05), a subtype which is most frequently observed in MSM in Israel [21]. On the other hand, the overall SBS-identified TDRM rate was similar in MSM (42.9%, 3/7) and non-MSM (57.1%, 4/7).

**Table 4.** Characteristics of patients with versus without NGS-identified or SBS-identified TDRM

	NGS			SBS		
Characteristic	TDRM, *n* = 25	No TDRM, *n* = 55	*P*-value	TDRM, *n* = 7	No TDRM, *n* = 73	*P*-value
Male, *n* (%)	21 (84.0)	43 (78.2)	0.76	7 (100.0)	57 (78.1)	0.33
Age, years, median (IQR)	32 (19–46)	30 (23–44)	0.61	45 (24–52)	30 (18–42)	0.04
Viral Load (log10 copies/ml), median (IQR)	4.9 (4.3–5.8)	5.8 (4.9–6.4)	0.03	5.6 (5.4–5.9)	5.5 (4.7–6.2)	0.55
CD4 (cells/mm3), median (IQR) (*n* = 50)	427 (431–616), *n* = 15	408 (315–576), *n* = 35	0.83	429 (327–567)	405 (317–585)	0.98
Risk group, *n* (%)						
MSM	15 (60.0)	32 (58.2)	NS	3 (42.9)	44 (60.3)	0.44
OGE	4 (16.0)	12 (21.8)	0.76	0 (0)	16 (21.9)	0.33
Other*	6 (24.0)	11 (20.0)	0.77	4 (57.1)	13 (17.8)	0.03
HIV subtype, *n* (%)						
B	16 (64.0)	32 (58.2)	0.24	7 (100)	41 (56.2)	<0.05
C	5 (20.0)	12 (21.8)	0.32	0 (0)	17 (23.3)	0.33
Other	4 (16.0)	11 (20.0)	0.27	0 (0)	15 (20.0)	0.33
Diagnosis at later years, 2008–2014	15 (60.0)	27 (49.1)	0.47	6 (85.7)	37 (50.7)	0.16
RT mutations, mean	12.6	9.7	0.03	8.5	8.3	0.83
PR mutations, mean	9	8.4	NS	6.4	7.6	0.27

MSM – men who have sex with men; OGE – originating in countries with generalized epidemics; TDRM – transmitted drug resistance mutations; PR – protease; RT – reverse transcriptase; SBS – Sanger-based sequencing; NGS – next-generation sequencing. *Other subtypes- A, AG/G,and F.

**Table 5.** Study population by years of diagnosis

Characteristic	2000–2007 (*n* = 37)	2008–2014 (*n* = 43)	*P*-value
Male, *n* (%)	26 (70)	38 (88)	0.04
Age, years, median (IQR)	31 (23–41)	30 (27–45)	0.37
Viral Load (log10 copies/ml), median (IQR)	5.9 (4.9–6.3)	5.2 (4.2–5.9)	0.47
CD4 (cells/mm3), median (IQR, *n*)	396 (319–606, *n* = 29)	431 (287–568, *n* = 21)	0.8
Risk group, *n* (%)			
MSM	20 (54.1)	27 (62.8)	0.49
OGE	14 (37.8)	2 (4.7)	<0.05
Other	3 (8.1)	14 (32.6)	<0.05
Subtype, *n* (%)			
B	22 (59.5)	26 (60.5)	1
C	14 (37.8)	3 (6.9)	<0.05
A	1 (2.7)	10 (23.3)	<0.05
G/AG/F	0	4 (9.5)	0.12
SBS TDRM, *n* (%)	1 (2.7)	6 (13.9)	0.12
NGS TDRM, *n* (%)	9 (24.3)	16 (37.2)	0.24

MSM – men who have sex with men; OGE – originating in countries with generalized epidemics; TDRM – transmitted drug resistance mutations; SBS – Sanger-based sequencing; NGS – next-generation sequencing.

### Characterization of recently infected HIV-1 individuals identified in 2000–2007 versus in 2008–2014

As significantly more of the newly diagnosed patients in 2008–2014 were defined as recently infected compared to 2000–2007, we studied the characteristics of the two populations (Table 5). More men (88%, 38/43, versus 70%, 26/37, *p* < 0.05) and more patients carrying subtype A (23.3%, 10/43 versus 2.7%, 1/37, *p* < 0.05) were identified among those recently infected in 2008–2014, as compared to the earlier period. In contrast, subtype C, which was identified in 21.2% (17/80) of individuals, was significantly (*p* < 0.05) more prevalent (14/37) among those considered to be recently infected in 2000–2007 compared to 2008–2014 (3/43). A higher rate of recently infected patients carrying any TDRM was observed during 2008–2014 (37.3% or 13.9%, as identified by NSG or SBS, respectively), in comparison to those diagnosed as HIV-1 positive in 2000–2007 (24.3% or 2.7%, as identified by NGS or SBS, respectively), but the differences were not statistically significant. While only one patient (1/37, 2.7%) identified before 2007 had multiple TDRM detected by NGS (PI and NRTI), eight patients diagnosed between 2008 and 2014 (8/43, 18.6%) had NGS-identified TDRM to two or more drug classes (*p* < 0.05).

### Long-term follow-up of and treatment outcomes in patients harbouring NGS-identified TDRM

Long-term follow-up information was available for 44% (11/25) of the patients with NGS-identified TDRM. Two of these eleven patients were not treated since initial diagnosis. The remaining nine were successfully treated and HIV-1 was not detected in samples taken within a median of 6 years (IQR 3–11) after initial diagnosis. Only two of these patients were treated with drugs that are expected to be affected by the identified TDRM. Both had K65R TDRM, known to be associated with reduced replicative capacity, and were infected with subtype C virus, supporting the hypothesis that this mutation can develop more easily in subtype C than in other subtypes [22]. Patient 26,203, harbouring K65R (1.5%), was treated with trizivir (abacavir, lamivudine and zidovudine) 5 years after initial diagnosis. K65R is known to reduce the activity of both abacavir and lamivudine, while increasing susceptibility to zidovudine [23]. Interestingly, the patient managed to respond well to these drugs and the HIV-1 viral load was undetectable. Patient 63,729, with the K65R mutation (2.5%) was successfully treated with truvada (TDF, emtricitabine and tenofovir disoproxil fumarate) and dolutegravir (DTG), a combined therapy that was initiated 3.7 years after the initial diagnosis. Interestingly, in five of these 11 cases, SBS performed on later samples failed to detect the previously identified minor TDRM, suggesting that viral variants with these mutations did not preferentially replicate.

## Discussion

This study assessed the prevalence of TDRM detected by NGS and SBS in early plasma samples from recently infected HIV-1 patients in Israel, between 2000 and 2014. Although only 30% of all the laboratory-defined, recently infected individuals diagnosed in these years were included in this analysis, their demographics accurately represent those of all 236 recently infected individuals identified between 2000 and 2014. As expected, significantly more non-synonymous amino acid substitutions and significantly more TDRM were identified by NGS. Studies comparing TDRM identified by NGS versus SBS, also demonstrated a higher number of NGS-identified pre-therapy resistance mutations as compared to SBS [24]. Here, the overall 8.8% prevalence of SBS-identified TDRM matched the 10.1% reported prevalence in treatment-naïve, recently infected HIV-1 individuals in Europe, with NRTI being the most frequently affected drug class, followed by NNRTI and less frequently (here, zero TDRM), PI [1,25]. Overall, 31.3% of the patients had NGS-identified TDRM. All PI TDRM were identified at low frequency (<7% of the viral population). In patients failing protease treatment, minority PI DRMs may suggest intermittent adherence to a boosted PI regimen and represent early events in resistance selection [26]. The source and the clinical relevance of the minor PI TDRM identified in this study in treatment naïve patients remains unclear. NRTI NGS-identified TDRM were the most frequent, observed in 26.3% of the patients. NNRTI NGS-identified TDRM were less common, but, compared to NRTI NGS-identified TDRM, more of the NNRTI TDRM dominated the viral quasispecies. Some of the low-frequency TDRM identified were APOBEC related, suggested, by others, to be artificial NGS-related variants [20]. TDRM identified at low frequency in the viral population may also result from fitness cost. While viral variants harbouring NRTI or PI TDRM are less fit and remain minority species that may be lost from the viral population with time, the higher prevalence of NNRTI TDRM reflects their relatively low influence on viral fitness. Therefore, variants with NNRTI mutations may better persist in the viral pool and interfere with future NNRTI treatments, especially as these drugs are considered to have a lower genetic barrier compared to NRTI and PI. Indeed, NNRTI mutations were shown to have the largest impact on drug susceptibility [1]. Taken together, even low-frequency, NGS-identified NNRTI TDRM should be considered in antiviral treatment selection.

Patients bearing NGS-identified TDRM had significantly lower HIV-1 viral load, a higher number of non-synonymous RT mutations and significantly lower mean prevalence of such mutations in the viral pool, most of which were below a prevalence of 5%. These results suggest that compared to HIV-1-infected persons without TDRM, patients with NGS-identified TDRM have a diverse population of high and low frequency viral variants, some of which are probably less fit compared to wild type. It has already been suggested that a viral population with high genetic diversity, is more likely to develop resistance mutations [27], as observed herein.

Proportionally, more HIV-1 seroconverters were diagnosed in recent years (2008–2014) compared to earlier years (2000–2007). This increase may be attributed to the continuous spread of HIV infections and to the introduction of improved diagnostic tools and higher awareness for HIV-1 over the years. The recently infected population identified in 2008–2014, included fewer subtype C and more subtype A1-infected individuals. These characteristics mirror the reduction in immigration from Ethiopia, characterized by a high subtype C prevalence, and the rise in immigration from the former USSR, including patients infected with subtype A1 HIV-1, during this time period [12]. Irrespective of the sequencing method used, the prevalence of TDRM among recently infected individuals was higher in 2008–2014 as compared to the earlier period, and significantly more patients diagnosed in this later period had multiple TDRM. Other studies report reduction in the overall prevalence of PI, NRTI and NNRTI TDRM with time [25,28] or stability in their prevalence [1], observations typically attributed to changes in treatment regimens which improved patient adherence. The inclining trend in TDRM prevalence identified in Israel, together with the steady increase in the number of overall new infections identified in 2008–2014 as compared to 2000–2007 (3144 versus 2686 HIV-1-positive individuals), warrants continuous monitoring of pretherapy drug resistance.

The fate of minority TDRM exclusively identified by NGS, is unclear as they may either revert to a wild-type phenotype or integrate into the host DNA and influence future treatment response [29]. Here, later time-point samples from patients with minor NGS-TDRM did not exhibit SBS-identified resistance mutations, suggesting that the TDRM did not emerge as major variants with time. Moreover, in the two cases treated with antivirals that could potentially be affected by the identified minor TDRM (NRTI K65R), treatment was successful. In a study conducted on 29 treatment-naïve patients, only one patient with low-frequency resistance mutations of relevance to the prescribed treatment, experienced virological failure [30]. Yet, as studies assessing the long-term clinical relevance of recently infected, low-frequency TDRM are rare, conclusions could only be drawn from larger studies in patients harbouring minor-frequency TDRM and exposed to therapy potentially affected by the identified transmitted mutations.

The high frequency (>20% of the viral population) NRTI M184V and T215S and NNRTI K103N mutations identified here in several cases, have already been reported to be primarily confined to recently identified patients diagnosed in more recent years [1]. The fate of each of these TDRM may be different. K103N is considered to have a limited effect on viral fitness [31] and, therefore, can be continuously transmitted as a major mutation that dominates the viral pool and impacts future therapy [32]. M184V, frequently observed in patients failing therapy [33], which is regarded as a mutation that reduces viral fitness more than any other NRTI mutation, may wane over time, likely as a consequence of reversion to the wild-type sequence [34,35]. The T215S mutation, restricted here to subtype B, was one of the most commonly observed TDRM in this subtype in the UK and was reported to persist in the viral quasispesies for many years [36].

We also observed several cases of RT E138 substitutions. Being polymorphic, this amino acid was not included in the TDRM surveillance list. However, studies do monitor this location, especially the E138A substitution inhibiting rilpivirine, whose prevalence has been shown to vary by geographical region and HIV-1 subtype [37]; in Europe, it was identified in 3.1% of the recently infected patients [1]. Here, six of the patients harboured E138 substitutions, three of whom had E138A. As these substitutions may be of clinical relevance, E138 mutations observed in recently infected individuals should be continuously monitored.

Taken together, our study further demonstrated that NGS can replace SBS for identification of TDRM and allows for better detection of low-frequency variants not detected by SBS, as already presented by others [26,30,38]. However, being more laborious and time-consuming, especially at low-volume settings, when higher costs may prevent its use, the overall benefits of the NGS system compared to SBS for baseline HIV-1 resistance analysis are questionable, especially as the fate of minor TDRM and their clinical relevance is still unclear.

The study had several limitations. Being conducted in Israel, it was limited by the total number of HIV-1 infections and of the recently infected individuals identified each year. Follow-up clinical information was available for only some of the patients with low-abundance TDRM, limiting our capability to draw conclusions on the long-term effects of such mutations. However, the type and prevalence of TDRM in Israel between 2000 and 2014 and utility of NGS to identify >1.5%-frequency TDRM in recently infected patients were demonstrated.

## Conclusions

With the increase in rates of TDRM in Israel between 2000 and 2014, resistance testing of treatment-naïve HIV-1 patients should continue. In this study, all PI and most of NRTI TDRM were identified at low frequency, while some of the NNRTI TDRM were highly prevalent in the viral pool, identified by both NGS and SBS and are likely to affect NNRTI-based therapy. Although low-frequency TDRM did not influence treatment response, follow-up of a larger cohort of individuals recently infected with HIV-1 TDRM is required to better establish this trend. Currently, the weight to place on minority TDRM in treatment decision-making processes remains unclear.

## List of abbreviations

NGS – next generation sequencing; NHRL – national HIV reference laboratory; PI – protease inhibitors; NNRTI – non-nucleoside reverse transcriptase inhibitors; NRTI – nucleoside reverse transcriptase inhibitors; PR – protease; RT – reverse transcriptase; SBS – Sanger based sequencing; TDRM – transmitted drug resistance mutations; LIA – Line-Immunoassay; MSM – men who have sex with men; OGE – originating in countries with generalized epidemics; PCR –polymerase chain reaction; RNA – ribonucleic acids

## Notes

To access the supplementary material to this article please see Supplementary Files under Article Tools online.
